# Characterizing microfluidic approaches for a fast and efficient reagent exchange in single-molecule studies

**DOI:** 10.1038/s41598-020-74523-w

**Published:** 2020-10-22

**Authors:** Julene Madariaga-Marcos, Roberta Corti, Silvia Hormeño, Fernando Moreno-Herrero

**Affiliations:** 1 Department of Macromolecular Structures, Centro Nacional de Biotecnología, Consejo Superior de Investigaciones Científicas, Madrid, Spain; 2 School of Medicine and Surgery, University of Milano-Bicocca, Monza, Italy; 3 Department of Materials Science, University of Milano-Bicocca, Milan, Italy

**Keywords:** Biophysics, Molecular biophysics

## Abstract

Single-molecule experiments usually take place in flow cells. This experimental approach is essential for experiments requiring a liquid environment, but is also useful to allow the exchange of reagents before or during measurements. This is crucial in experiments that need to be triggered by ligands or require a sequential addition of proteins. Home-fabricated flow cells using two glass coverslips and a gasket made of paraffin wax are a widespread approach. The volume of the flow cell can be controlled by modifying the dimensions of the channel while the reagents are introduced using a syringe pump. In this system, high flow rates disturb the biological system, whereas lower flow rates lead to the generation of a reagent gradient in the flow cell. For very precise measurements it is thus desirable to have a very fast exchange of reagents with minimal diffusion. We propose the implementation of multistream laminar microfluidic cells with two inlets and one outlet, which achieve a minimum fluid switching time of 0.25 s. We additionally define a phenomenological expression to predict the boundary switching time for a particular flow cell cross section. Finally, we study the potential applicability of the platform to study kinetics at the single molecule level.

## Introduction

Single-molecule techniques such as magnetic tweezers (MT), optical tweezers (OT) or fluorescence microscopy employ flow cells to confine the biological molecules of interest in a reduced environment, normally under close to physiological buffer conditions^1^. Additionally, it is useful to allow the exchange of reagents in the experiment. This is crucial in reactions that need to be triggered by ligands, i.e. helicases which need ATP^2–5^, or require sequential addition of proteins in complex DNA–protein interaction reactions involved in processes such as DNA replication, transcription, DNA repair or chromosome organization^6–10^.

**Figure 1:**
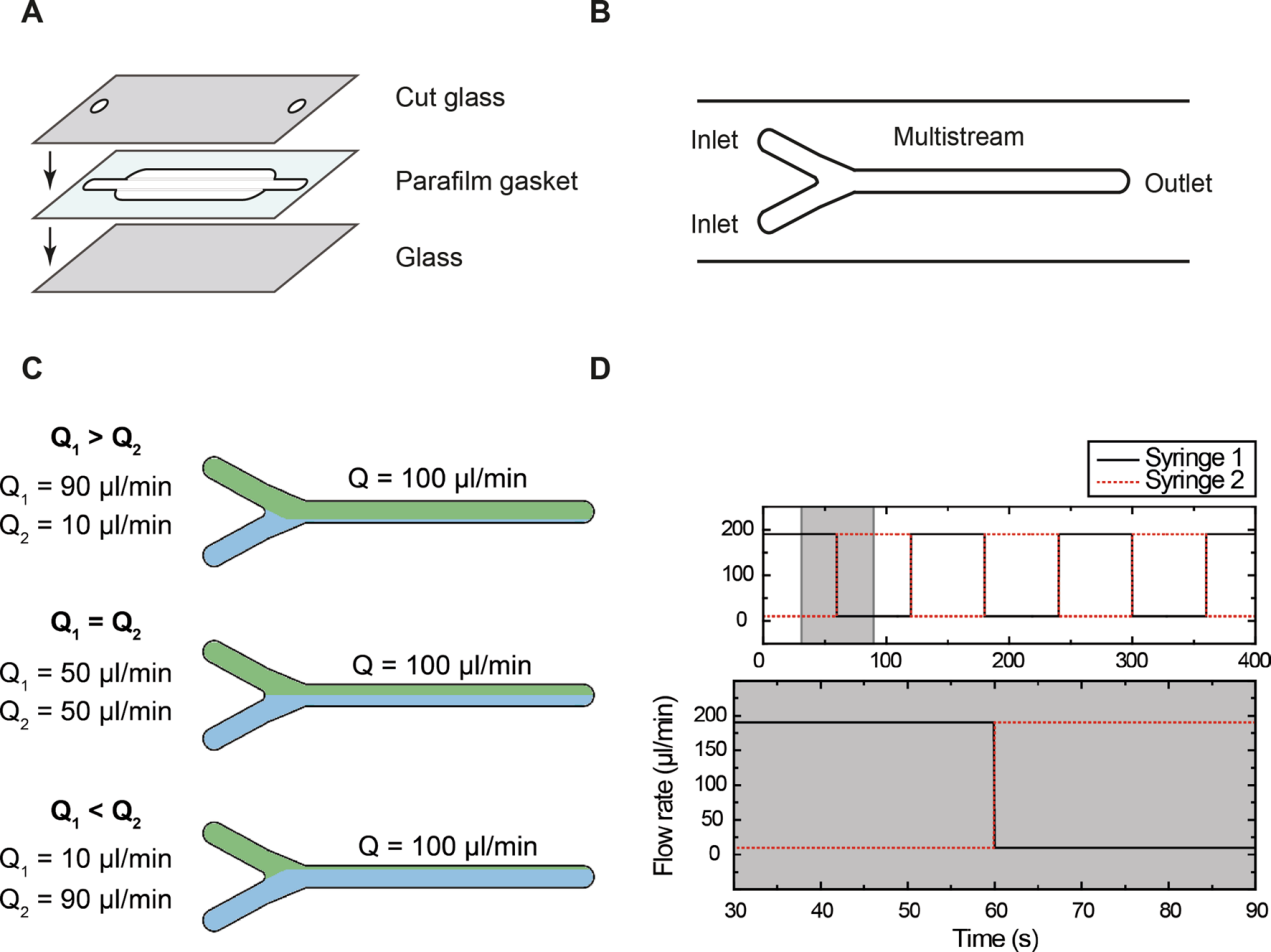
Approach for multistream laminar flow microfluidics. (**A**) Scheme of the simplest flow cell employed in single-molecule experiments. It is typically composed of two coverslips (one of them drilled) and a parafilm or double-sided tape gasket (composed of one or two layers). The coverslips sandwich the gasket allowing to flow reagents in and out. (**B**) Multistream microfluidic cells with two inlets and one outlet. (**C**) Fluids in a multistream laminar flow never mix and the boundary between them can be shifted altering their relative flow rates^1^. (**D**) Boundary shifting is achieved implementing automated flow rate variations called “flow rate profiles”.

Simplest flow cells are normally home-fabricated using two coverslips or microscope slides (made of glass or quartz) with a channel in between. The channel can be created using a gasket made of paraffin wax (parafilm) or double-sided tape (Fig. 1A)^11^. The volume of the flow cell can be controlled by modifying the dimensions (length, width and depth) of the channel and the number of gasket layers, as well as the gasket material, i.e. double-sided tape, which is thinner, or parafilm, which is thicker^12^. Commercial solutions are available for sophisticated applications, but custom-built flow cells are the most widespread approach. Furthermore, these give versatility in the design and fabrication procedure. These cells are normally limited to a number of experiments and are then discarded. The affordability of materials as well as the simple fabrication procedure makes them worthless to recycle, with the exception of those made of quartz slides^13,14^.

Reagents can be then introduced in different ways including pipetting^15^ or sucking with a piece of paper^16^. However, these methods can generate uncontrolled and turbulent flow-rates that could shear DNA tethers. This can be avoided by introducing reagents in a controlled manner using a syringe pump^3,17,18^, which is the most common method. In some cases, flow cells are afterwards sealed to perform the experiment^16^. However, the standard experimental scenario maintains at least an inlet and an outlet open for reagent exchange^12,19,20^.

While this system, comprising a single-channel flow cell and a syringe pump, is useful and precise enough in a vast variety of single-molecule assays^21–25^, it has a main limitation. A fast exchange of reagents is not possible without working under very high flow rates, thus disturbing the biological system in the flow cell. Furthermore, the velocities employed not to perturb the system usually lead to the generation of a reagent gradient in the flow cell^3,12^. This has been particularly important in studies involving helicase ATP hydrolysis^2,3,5^, where the ATP gradient leads to a gradual start of the translocation by the enzyme, with a slower velocity right after injection. For precise time-resolved measurements it is thus desirable to have a fast exchange of reagents with minimal diffusion. In the context of fluorescence experiments it is also interesting to be able to rapidly and/or briefly expose a sample to the fluorescent probe^26^. For instance, intercalating agents generate free radicals upon excitation, which damage nucleic acids^27^. In many fluorescence measurements, these reagents are employed to visualize DNA, then washed away and subsequently substituted by fluorescently labelled proteins^28,29^. Altogether, a precise control of rapid buffer exchange is a considerable improvement for such applications.

A widely-explored solution for fast buffer exchange is the implementation of multistream laminar microfluidic cells consisting of at least two inlets and one outlet (Fig. 1B)^12,30,31^. By definition, a flow is laminar when it follows the contour lines of the channel confining it^32^. Under laminar flow conditions, the Reynolds number of the system (which depends on the fluid, the velocity and channel dimensions) must be below 2000. In a previous work, we proved that the flow is always laminar in the case of conventional MT flow cells^33^. In this scenario, because of the nature of laminar flow, a fast exchange of reagents can be achieved while diffusion is minimized^12,32^. Under laminar flow conditions, parallel streams of a fluid running through a single channel will flow parallel and will not mix^12^, making laminar flow cells interesting for single-molecule applications. This permits an accurate and fast exchange of reagents in our proposed two-inlet flow cells and keeps the setup simple, making minor modifications to a classical MT flow cell holder.

There are several ways to rapidly expose a sample to different reagents. Once a set of parallel streams is achieved, the sample could be moved from one stream to another as it is done in optical tweezers (see for example^34^). However, this is not possible in MT or TIRF because these techniques rely on the sample being attached to the surface. Taking this requirement into account, an alternative solution is to shift the boundary between the streams to expose the sample to different fluids, as it is depicted in Fig. 1C.

In this work, the optimization and characterization of homemade microfluidic cells for sophisticated and fast reagent exchange will be discussed. We will show that a thorough design led us to the fabrication of optimized flow cells with smaller channel dimensions and higher linear velocities, with minor modifications to the flow cell holder. The flow cell suitability for single-molecule experiments has been addressed by means of TIRF measurements and magnetic tweezers experiments. We additionally show that the time for boundary switching between reagents can be predicted based on the geometry of the flow cell.

## Results

### Principles of multistream microfluidics

The basis of the fast reagent switching relies on laminar flow theory, stating that two parallel streams will not mix^12^. Nevertheless, the position of the boundary between the streams can be altered by controlling the relative flow rate of each stream. As shown in Fig. 1C, if the two inlets have the same flow rate, the boundary will be placed in the middle of the channel. If the inlet flow rates are altered but the integrated flow rate in the central channel is maintained, such as one inlet being 90% of the flow rate and the other one being 10%, the boundary will be shifted towards the low flow rate inlet. If the flow rates in the inlets are inverted, the boundary will shift towards the other inlet. This allows exchanging the buffer, protein or ligand in the flow cell without the need of moving the sample; thus conferring it suitability for MT-TIRF experiments.

Several commercial fluidic systems can be employed for a fast alternation of flow rates. We decided to choose conventional syringe pumps to directly control the velocity of the inlet flows, which is a critical parameter to alter the boundary. Specifically, we implemented a pair of computer controlled syringe pumps (neMESYS Low Pressure module, Cetoni). We employed a pair of glass syringes (1 ml PTFE-PEEK thread, Setonic 2624016 and 10 ml PTFE-PEEK thread, Setonic 2624076) and PTFE tubing to connect them to the flow cells (PTFE 1.6 OD, Cetoni 701175). Alternative solutions controlling the pressure of a liquid reservoir; thus the velocity of the outlet, were also considered^35,36^. These have been widely employed by other authors in multiple studies^37–40^. However, a direct control of the flow velocity was more suitable for our application, where molecules are fixed to a glass surface, over a pressure-based system for several reasons. To start with, pressure leaks would lead to changes in velocity, which would disturb the laminar flow boundary^40^. In addition, control over velocities is straightforward in our chosen fluidic system but would require converting pressure data into the corresponding velocities in a pressure-based system. Pressure-based systems are preferred for optical tweezers approaches where the user moves the optical traps through the different laminar streams and potential pressure drops that equally affect all fluid channels are not critical^41^. Being the applied flow rate (and thus the linear velocity) the essential parameter to alter the boundary between the streams, syringe pumps were our preferred approach.

In this work, we used two kinds of syringes, with volumes of 1 ml and 10 ml, and changed the flow rate following square waves with maximum rates of 90% of the central channel flow rate and minimum rates of 10%, as shown in Fig. 1C, for an instantaneous shifting of the boundary towards one end or the other. The system has a temporal resolution of 100 ms, as provided by the manufacturer (Fig. 1D). Finally, for experiments involving MT tracking it was necessary to linearly increase the velocity at the beginning of the experiment to keep a proper tracking of the bead.

### Geometric considerations for flow cells with faster boundary shifting

**Figure 2:**
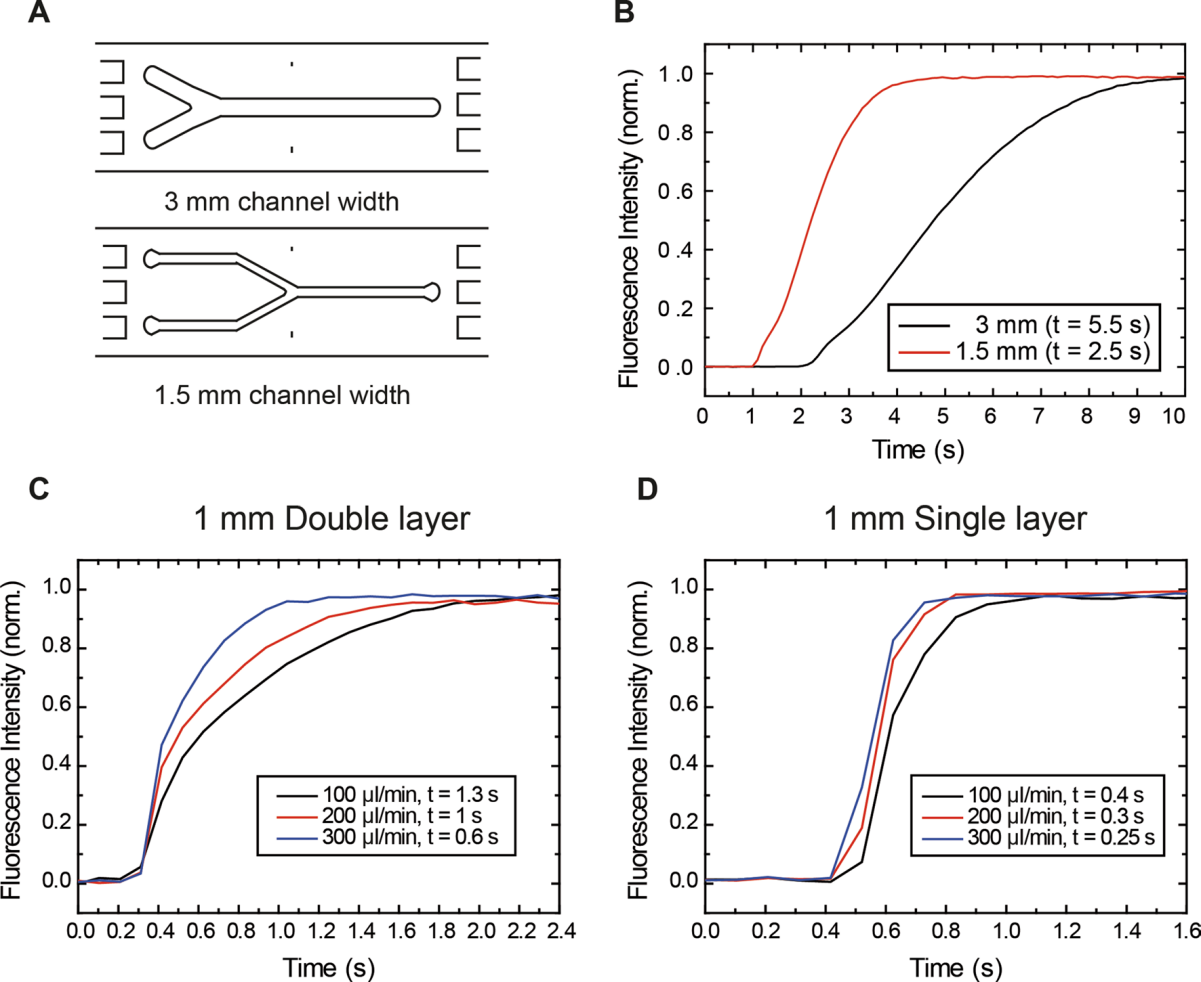
Design and boundary exchange performance of optimized flow cells for different flow rates. (**A**) Initial multistream flow cell design employed in previous studies^42^ (top) vs. optimized version employed in this study (bottom). (**B**) Boundary shifting using 1 mM fluorescein demonstrated that the boundary exchange time is lower in case of narrower flow cells and closer to the inlet (red) compared to previously employed wider flow cells far from the inlet (black). (**C**) Boundary shifting for different flow rates (100–300 µl min^−1^) for double parafilm layer flow cells. (**D**) Boundary exchange for different flow rates (100–300 µl min^−1^) for single parafilm layer flow cells.

We have previously reported the use of double-inlet flow cells. However, conventional flow cells for single-molecule experiments are in the order of several millimeters wide (Fig. 2A, top panel) which limits the boundary shifting times to several seconds^42^.

Previous computational studies on miniaturized flow cells have characterized the boundary shifting response time using the Taylor-Aris model^43–45^. This model describes the boundary shifting considering a variation of the concentration (due to reagent exchange) as a function of time $c\left(t\right)$ considering both diffusion and convection (Eq. 1):

1 $$c\left(t\right)={c}_{0} \frac{1}{2} \left(1+Erf\left[\frac{y-vt}{2\sqrt{tk}}\right]\right)$$

where ${c}_{0}$ is the concentration of fluorophores, $v$ is the average linear velocity, $k$ is the effective diffusion coefficient $\left(k=D+ \frac{{av}^{2}}{210D}\right)$ and $y$ the distance from the junction. $v$ and $k$ should change with flow rates and between different cross sections while $y$ should remain unchanged among the same flow cell.

This theory is valid when $y$ >> ${y}_{0}$ from the two-inlet junction, being ${y}_{0}$ defined as (Eq. 2):

2 $${y}_{0}= \frac{{a}^{2} {v}_{0}}{288 D}$$

where ${v}_{0}$ is the velocity of the fluid, $a$ the height of the channel and $D$ the diffusion coefficient.

Equation 1 shows that the key parameters affecting the boundary shifting performance are $y$, $a$ and $v$. These parameters should be considered for an efficient design of optimal flow cells. First, in terms of $y$, the closer to the inlets junction, the faster the reagent exchange occurs. Flow cells should be redesigned such that the junction of the inlets matches the microscope objective. Secondly, reducing the cross-section dimensions (height, width) of the flow cell leads to higher linear velocities and a faster boundary shifting. This also accounts for a smaller channel height, $a$. Finally, employing higher flow rates would also lead to higher linear velocities, thus, to a faster reagent exchange. However, this is experimentally limited by the pressure on the tubing and the flow cell, and should be carefully taken into account.

Considering these facts, we modified the design employed in a previous study^42^ (with a channel width of 3 mm, being $y$ ≈ 15 mm) to a new one with 50% reduced dimensions and a displaced channel junction (with a channel width of 1.5 mm, being $y$ ≈ 1–2 mm) (Fig. 2A, bottom panel). To experimentally measure the boundary shifting and in order to compare the flow cell performance, we employed a 1 mM fluorescein solution while flowing at 200 µl min^−1^. To quantify the switching response between fluids, we defined the switching time t_switch_ as the time to go from 10% intensity to 90% intensity^33,43^. It can be readily seen from Fig. 2B that the switching time was considerably decreased for the narrow flow cells under the same flow rate conditions, dropping from 5.5 to 2.5 s. Considering this improvement, we aimed to perform a thorough study on how linear velocities affected the boundary shifting response time.

To characterize the boundary shifting time at different linear velocities, we employed 1 mm wide flow cells, which was the narrowest width we could achieve with our parafilm gasket approach. We fabricated double and single parafilm layer flow cells (100 µm height for 1 parafilm layer and 200 µm height for 2 layers) and employed the fluorescein experiment described in the section above. Taking advantage of the simulations described in^43^, we focused all the measurements in the proximity of the junction between channels ($y$ ≈ 1–2 mm) to achieve the fastest possible boundary shifting. We decided to explore the switching time as a function of different flow rates, ranging from 100 µl min^−1^ to 300 µl min^−1^. Employing higher flow rates was attempted but it resulted in leakage due to the high pressures in the tubing and connections.

Response times of the boundary shifting in double parafilm layer flow cells (Fig. 2C) exhibited a rather expected behavior, with higher flow rates showing a faster reagent exchange. The t_switch_ value went down to 0.6 s for a flow rate of 300 µl min^−1^. To study higher linear velocities, we employed single parafilm layer flow cells (Fig. 2D) in which the boundary changed even faster, achieving response time of 0.25 s in the case of 300 µl min^−1^. This demonstrates that our approach can achieve response times of less than 0.5 s employing reasonable flow rates and appropriate channel dimensions for single-molecule experiments.

### Predictions on boundary exchange based on flow cell geometry

The shape and dimensions of the flow cell, i.e. its cross section, are the main features involved in a fast boundary shifting and the easiest parameters to tune, along with the flow rate. To characterize the effect of the channel dimensions in boundary shifting, we fabricated several flow cells (Supplementary Fig. 1), with different heights and widths, using the 2-inlets and 1-outlet design. These flow cells had channel widths of 1, 1.5 and 2 mm and channel heights of 100 µm (in the case of 1 parafilm layer) and 200 µm (in the case of 2 layers).

We performed the boundary shifting experiment using 1 mM fluorescein as described in the previous sections employing a range of flow rates (7 µl min^−1^ to 200 µl min^−1^) indicated in Supplementary Table 1. We fitted our fluorescence vs. time signals to the Taylor-Aris model employing $v$, ${y}_{0}$, $a$ and $D$ as free parameters. Results for the Taylor-Aris fitted data are reported in Supplementary Table 2. The reported parameters from the fit are within the expected order of magnitude of the theoretical reported values considering that v_0_ = 160 µm s^−1^ is the velocity of the fluid for a 100 µl min^−1^ flow rate, $a$ = 100–200 µm the height of the channel and $D$ = 4.90 10^–10^ m^2^ s^−1^, the diffusion constant for fluorescein. This leads to ${y}_{0}$ ≈ 0.004 mm, thus fulfilling the condition $y$ > > ${y}_{0}$.

**Figure 3:**
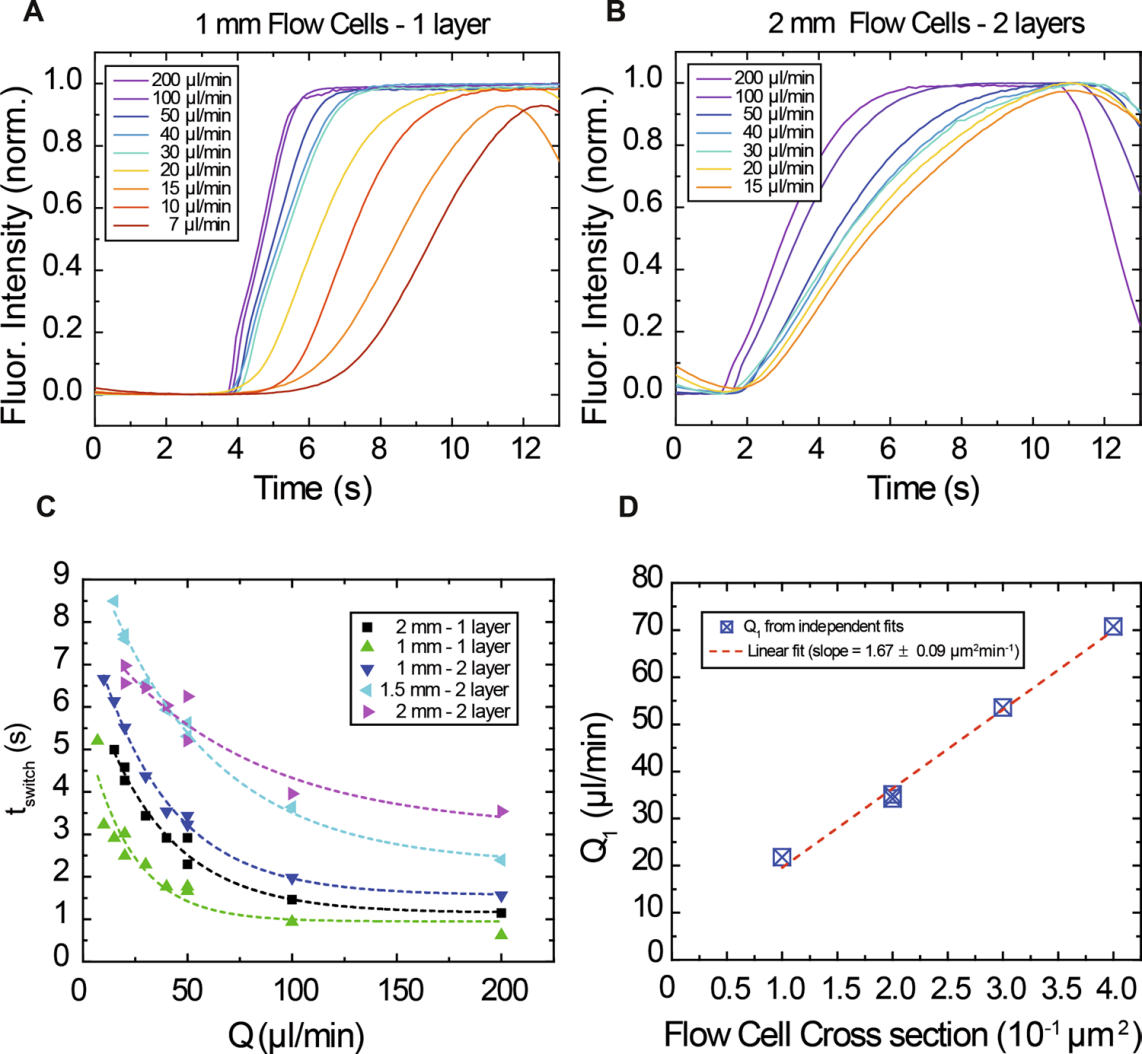
Boundary shifting time prediction for a given flow cell geometry. (**A**) Measured changes in fluorescence intensity at different flow rates for 1 mm wide single layer flow cells. (**B**) Measured changes in fluorescence intensity at different flow rates for 2 mm wide double parafilm layer flow cells. Note that slower flow rates as well as bigger cell dimensions (and thus bigger cross-sections) give a slower reagent exchange time. (**C**) Correlation between the applied flow rate Q and the switching time t_switch_ for different flow cell geometries. Results can be fit to simple exponentials, being the exponent the fitting parameter Q_1_. (**D**) The fitting parameter Q_1_ allows us to predict the boundary shifting time for a particular flow cell cross section, facilitating flow cell design to achieve the desired reagent exchange.

Figure 3A,B report the measured changes in fluorescence intensity at different flow rates for 1 mm wide single layer flow cells and 2 mm wide double layer flow cells, respectively. These two flow cells show the biggest differences in boundary shifting response time due to the high difference in cross section. Additional data for the rest of the flow cells is shown in Supplementary Fig. 2.

Aiming to unravel a correlation between flow cell dimensions and the response time t_switch_, we plotted the obtained t_switch_ values as a function of the flow rate for different flow cell geometries (Fig. 3C). Interestingly, the resulting curves could be nicely fitted with simple exponentials with one single fitting parameter Q_1_ (Eq. 3):

3 $${t}_{switch}\left(Q\right)= A {e}^{\frac{Q}{{Q}_{1}}}+{t}_{0}$$

${t}_{0}$ was left as a free parameter but was < 10^–4^ s in all the studied cases. This model is purely phenomenological but resulted very useful describing our experimental data. Furthermore, we could relate ${Q}_{1}$ to the flow cell cross section, which resulted in a simple linear relationship with a slope of ≈ 1.67 µm^2^ min^−1^ (Fig. 3D). Notice that 1 mm wide and 200 µm high flow cells as well as 2 mm wide and 100 µm high flow cells (which have exactly the same cross section) give precisely the same ${Q}_{1}$ value. Importantly, the linear relationship of the fitting parameter ${Q}_{1}$ with the cell cross section allows us to predict the boundary shifting time for a particular flow using Eq. 3, facilitating flow cell design to achieve the desired reagent exchange time. The relationship between the shifting time and the flow cell cross section for flow rates of 15, 50 and 200 µl min^−1^ are shown in Supplementary Fig. 3.

We note that our model is limited by the specific dimensions of our flow cells and the materials employed in the tubing and syringes. Nevertheless, the materials we use here are standard to the single molecule community and our model still provides a simple yet useful result. The employment of different materials would most likely give phenomenological parameters different to those presented here, and more sophisticated modelling would be required to describe this in detail. Specifically, one could describe the behavior of the system taking advantage of equivalent circuit theory as suggested by other authors^46^. This analysis would provide a new level of details but it is beyond the scope of this work.

In summary, our phenomenological model succeeded in explaining our experimental boundary shifting data and it permits predicting the boundary shifting response time for a specific flow cell design, when employing standard materials for the single-molecule community.

### Performance in flow-stretched DNA experiments

**Figure 4:**
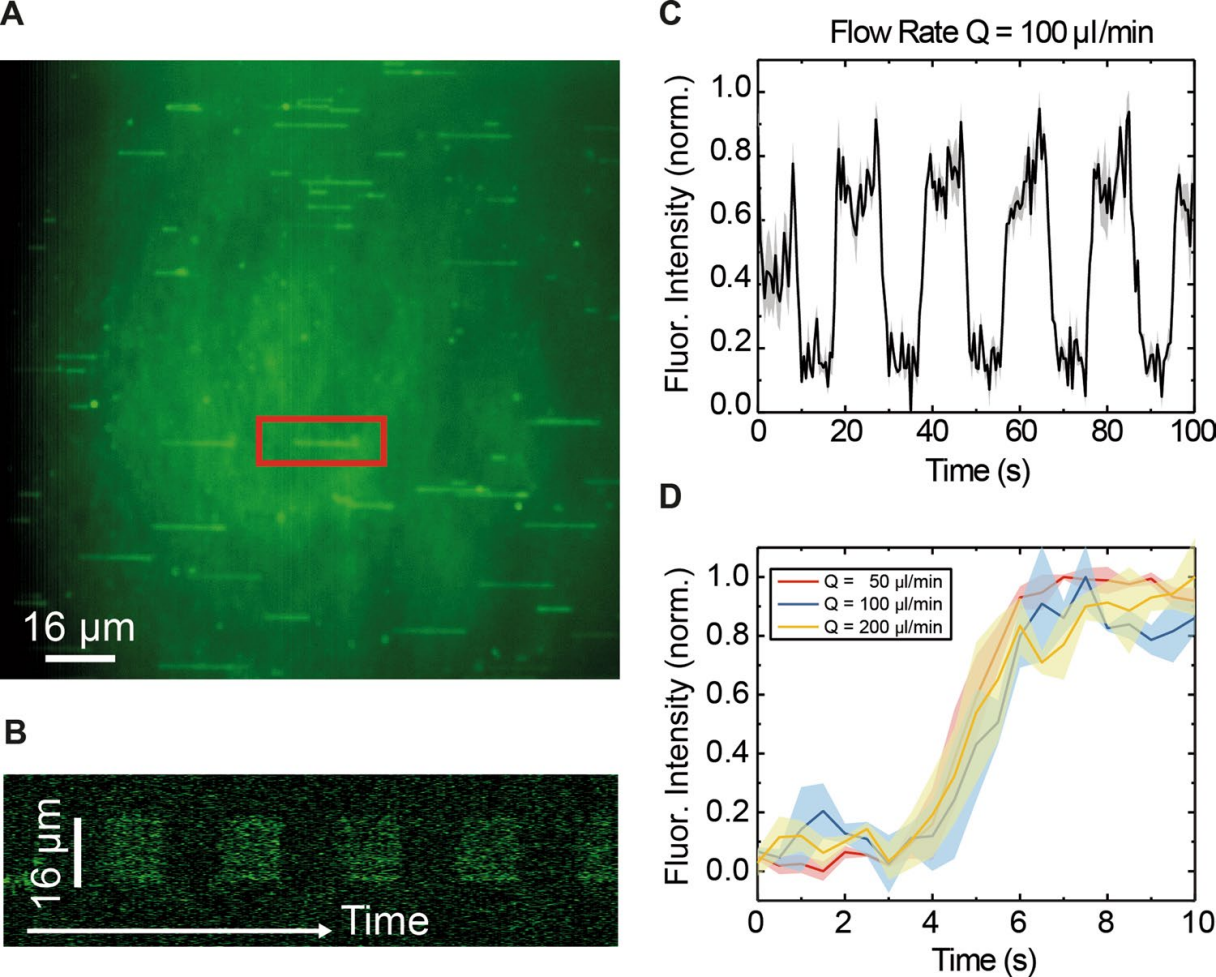
Boundary exchange measurements using DNA molecules labelled with Sytox Green. (**A**) Typical field of view with 48 kbp long linear λ DNA molecules stretched under the presence of 100 nM Sytox Green. (**B**) The kymograph on the bottom shows the alternation between buffer and Sytox as the intensity on one DNA molecule (red square) increases and decreases. (**C**) Averaged integrated fluorescence intensity as a function of time for three DNA molecules, showing an increase when Sytox binds and a decrease when it unbinds. (**D**) Zoomed region of the fluorescence intensity as a function of time upon the introduction of Sytox at different flow rates, showing that the increase in fluorescence signal due to Sytox binding remains the same.

After having characterized different flow cell designs, we employed the narrowest one (1 mm wide—1 parafilm layer) to characterize the behavior of DNA under the influence of an applied flow. To visualize the DNA under TIRF illumination we employed the intercalating agent Sytox Green, widely employed in DNA-fluorescence assays^27^. Sytox dyes exhibit a large (> 1000-fold) fluorescence enhancement upon DNA binding^27,47–49^. Moreover, Sytox Green binds DNA rapidly and with high affinity, has a good signal-to-noise ratio even at low concentrations, exhibits a low photobleaching rate and induces lower light-induced DNA degradation compared to other intercalating dyes. In this work, we employed Sytox at a concentration of 100 nM and λ DNA molecules of ~ 16 µm (48 kbps). Different flow rates were applied to the sample, ranging from 50 to 200 µl min^−1^ (Supplementary Fig. 4). A representative experiment at 100 µl min^−1^ is shown in Fig. 4. We were able to visualize many DNA molecules stretched under the flow exhibiting the expected extension (Fig. 4A).

We employed our microfluidics platform to alternate between buffer and buffer containing Sytox Green. The fluorescence intensity along the DNA molecule increased in the presence of Sytox and decreased when buffer was introduced (Fig. 4B, and 4C). This can be clearly seen in the kymograph shown in Fig. 4B. Moreover, the dynamics of the system DNA:Sytox could be easily solved assuming that the concentration of Sytox remained constant during the injection phase. This was a reasonable assumption, since there was an excess of dye compared with DNA and our measurements were significantly longer than the boundary shifting time.

We measured a 2 s exchange time independently of the flow rate by considering intensity changes from 10 to 90%, as previously mentioned (Fig. 4D). This value is consistent with other works reporting slow kinetics (50 s saturation time at 10 nM)^27^. This process is over the limit of the boundary shifting response time (with a reported value of 0.4 s for a flow rate of 100 µl min^−1^), which proves that our platform is suitable to study kinetics. This is further supported by kinetic studies using our microfluidics setup carried out in our previous work^42^.

### Performance in magnetic tweezers experiments

**Figure 5:**
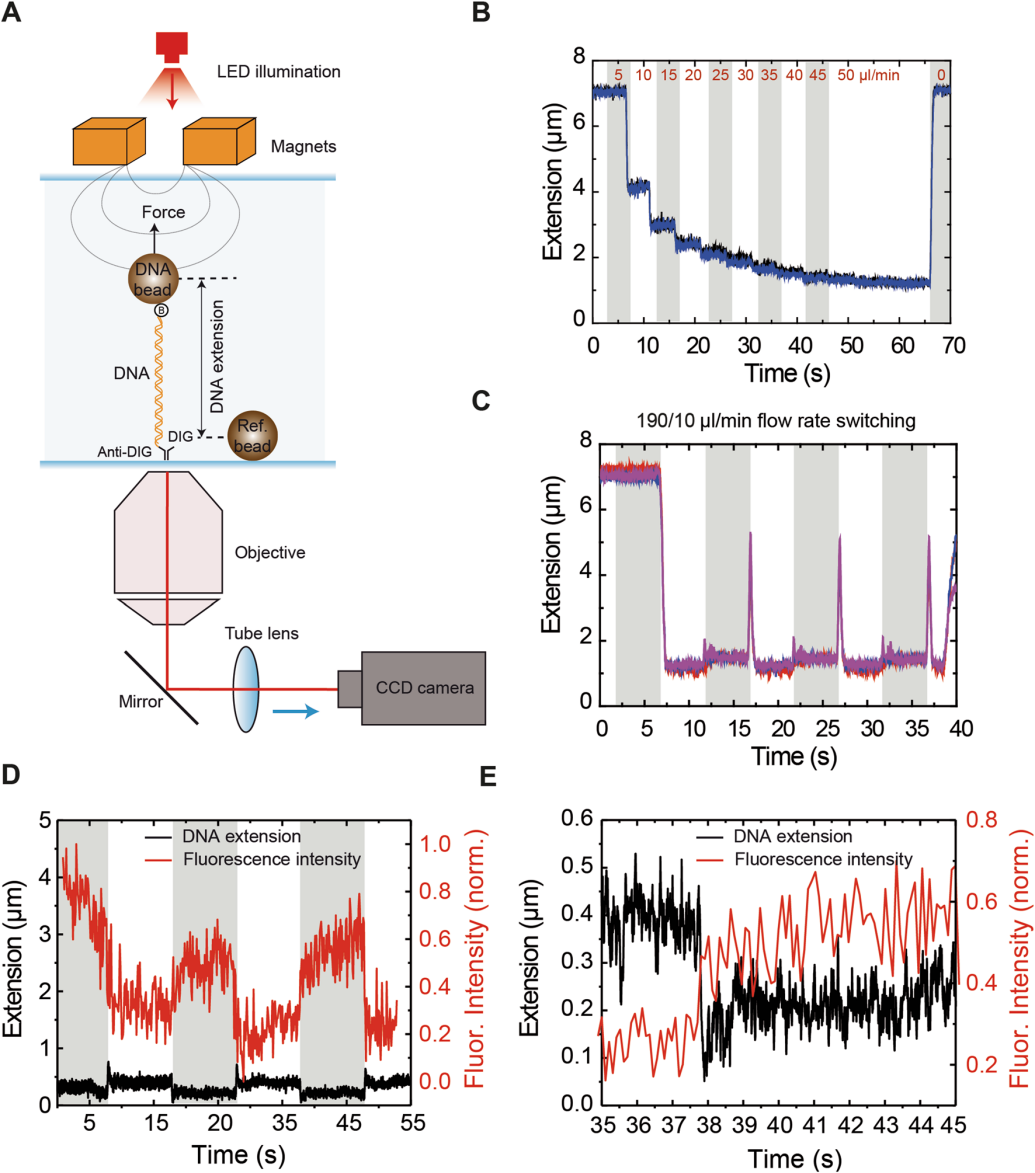
Boundary exchange assessed by Magnetic Tweezers experiments. (**A**) Magnetic Tweezers overview. They comprise a pair of magnets and an inverted optical microscope. In a flow cell, a DNA molecule tethers a superparamagnetic bead to a glass surface. DNA extension is calculated by comparing the vertical positions of a DNA bead that tethers the DNA and a reference bead attached to the glass surface. Vertical bead positions are calculated by comparing the pattern of diffraction rings they generate with a calibration look-up-table graph produced by moving the optical objective with a piezo stage. Force acting on the bead is calculated from the Brownian fluctuations of the bead as described in^4^. (**B**) DNA extension of 24 kbp long DNA molecules under 1 pN force was drastically affected (decreased) by the application of flow in 1 mm-wide flow cells. (**C**) Boundary shifting by altering the relative flow rates in the syringes induced a mechanical perturbation on the bead which is measurable by MT. Different sizes of the measured peaks are due to a difference in the syringe volume. (**D**) Correlated MT and TIRF data using DNA tethers in the presence of 50 nM Sytox Green. The changes in fluorescence correlate with the mechanical perturbations of the syringe. Furthermore, the force of the flow overcomes the magnetic force in this configuration. (**E**) Zoomed region of **D** showing the correlation between the boundary shifting, the mechanical perturbation of the syringe and the increase in fluorescence intensity.

We additionally characterized our flow cell performance using magnetic tweezers (MT) (Fig. 5). A standard MT setup comprises a pair of magnets coupled to an inverted microscope as depicted in Fig. 5A ^50^. By attaching a superparamagnetic bead to the free end of a DNA molecule attached to a glass surface, force and torque can be applied to single DNA molecules. Using custom-written algorithms, the extension as well as the force applied to the DNA can be tracked in real time. We studied the effect of increasing flow rates on 24.5 kbp long DNA molecules under a 1 pN force. Experiments were performed with 1 µm beads and in a 1 mm—1 parafilm layer flow cell. As pictured in Fig. 5B, even low flow rates of 10 µl min^−1^ drastically affect the DNA tethers by pushing the bead towards the surface of the flow cell, reducing their vertical extension. Furthermore, aside of altering the extension measurements, switching the syringe flow rates lead to syringes mechanically perturbing the traces by the introduction of periodic spikes (Fig. 5C). However, the differences in the amplitude of the spikes arise from the different volumes moved by the 10 ml and 1 ml syringe. In this experiment, we employed the same buffer in both syringes as a proof-of-concept. Employing the same flow cell dimensions and flow rates as in the fluorescence experiment has a drastic effect in the DNA attached to the bead, compared to the flow-stretched DNA employed in TIRF experiments. These measurements indicate that the flow generated in such miniaturized flow cells bend the DNA-bead system toward the surface in the direction of the flow.

To demonstrate a possible application of MT and our microfluidic setup, we repeated the experiment including 50 nM Sytox Green (Fig. 4) with magnetic beads and applying a magnetic force of 1 pN, and alternating flow rates of 180 and 20 µl min^−1^. As expected, the force applied by the flow overcomes that of the magnet (Fig. 5D) and the periodic spikes due to buffer exchange were detected. Furthermore, the fluorescence data captured by the TIRF microscope show how the mechanical pulses of the syringes perfectly correlate with the changes in fluorescence due to the exchange between Sytox and buffer (zoomed area in Fig. 5E). The mechanical perturbation of the syringe, in the order of 100 ms, is consistent with the response time given by the manufacturer. Additionally, the measured exchange of Sytox on the DNA is consistent with the kinetics reported in the aforementioned experiment. Altogether, this experiment is useful to illustrate the implementation of our microfluidic device in MT. However, we note that this experiment has the additional complication that the force applied to the DNA led to a faster degradation of the duplex in the presence of intercalating agents. On the other hand the force that is constant along the DNA molecule (in contrast with stretch flow experiments) can be estimated as previously done (see Supplementary Methods)^33^. Extension versus flow data could be correlated with the applied flow using previously taken force-extension curves and fitting to an inverted Worm-like chain model (Supplementary Fig. 5A). The obtained forces (Supplementary Fig. 5B) were considerably higher than those measured in conventional flow cells (4 pN compared to 1.5 pN), which was expected due to the applied higher linear velocities. This demonstrates that flow cells with reduced dimensions allow the application of higher forces in flow stretch experiments using beads.

## Discussion

We implemented a device based on multistream laminar flow technology that allows a fast exchange between reagents that minimizes diffusion. This device is based on two computer-controlled syringe pumps and home-fabricated two-inlet flow cells. Our device operates with a time resolution of 100 ms and has a minimized lateral diffusion compared to the width of the central channel. We have also proved that the switching of the syringes is correlated with boundary shifting by MT tracking and fluorescence measurements using fluorescein and Sytox Green.

The implementation of narrow flow cells (1 mm) for flow stretching measurements allowed a rapid exchange of reagents without affecting the behavior of the stretched molecules. The fabrication of the flow cells was identical to those conventionally employed in MT experiments and we observed that the elimination of accidental bubbles was facilitated by the high achievable linear velocities. Moreover, these flow cells were extremely versatile and could potentially be employed in both lateral and vertical MT experiments as well as in TIRF-based assays (or in combined MT-TIRF measurements).

The switching time t_switch_ between two different solutions was lowered to less than 2 s in a remarkable range of applied flow rates (with less than 1 s for Q > 100 µl min^−1^). We proved that the flow cell dimensions can be optimized to achieve exchange times of less than 0.5 s. For the 3 mm-wide design the t_switch_ was approximately of 4 s at Q = 200 µl min^−1^^42^; the reagent exchange of the flow cells presented in this study was at least 4 times faster than the previous ones.

Importantly, we demonstrated that the boundary switching response time t_switch_ can be correlated to the applied flow rate using one single parameter. This parameter Q_1_ can be related to the flow cell dimensions leading to a more efficient design of flow cells to meet specific experimental needs.

The Sytox-labelled DNA results reported here represent a good evidence for more sophisticated designs of stretched DNA experiments. They constitute a robust proof-of-concept experiment that our microfluidics platform can be applied to study kinetics, consistent with our previous report^42^. The same experimental setup (two inlets and one outlet) could be easily employed for testing DNA:protein binding, both in the presence of fluorescently-labelled proteins and/or intercalating dyes, or to visualize DNA superstructure as supercoiling as described before^47^. Our setup has already proven to be suitable for studies involving labelled proteins^42^, and could straightforwardly be used with Quantum Dots to obtain a co-localized two channel fluorescent image of, for example, protein and Sytox-stained DNA.

Our magnetic tweezers measurements showed that we can accurately measure the force applied to the DNA even under flow stretch. However, using such narrow channels, measurements can be hindered by the drag on the bead and the mechanical perturbation of the syringes. We showed that in MT and MT-TIRF combined measurements the force is mainly dominated by the high linear velocities of the flow. Correlated experiments proved that our boundary is shifted in a fast and accurate way, yet the force exerted by the flow surpasses that of the magnets. Finally, by monitoring single DNA extensions and using individual force extension curves, we estimated the forces exerted on a bead in flow-stretch experiments as done in a previous work^33^. We showed that the measured forces were sensibly higher than the ones previously reported with the previously employed 3 mm-wide flow cell design^33^ going from less than 1.5 pN to 4 pN.

In this context, more complex and miniaturized flow cells can be fabricated using polydimethylsiloxane (PDMS) chips, but these require less affordable materials and more sophisticated fabrication procedures^17,18^. Furthermore, we believe our approach is more suitable for surface-based single-molecule studies (like MT and TIRF) because glass can be easily functionalized with a variety of chemical attachments for binding biological molecules (like biotin and antidigoxigenin).

In conclusion, we demonstrated the crucial role of both the shape and dimensions of flow cells for single-molecule experiments and we proposed an optimized flow cell design, which allowed, in the proximity of the channel junction, to achieve a fast boundary exchange and, if necessary, to apply an external force comparable to the typical range of MT-based setups. Our proposed method was easy to implement and cheaper than commercially available products or homemade PDMS microfluidic devices employed in other studies^43^. The reduction of the volume was also essential to limit the quantity of solutions, ligands and sample necessary to perform the experiment. Moreover, the flow cells described in this work could be completely customized. The glass surface could be cleaned and functionalized in several different ways (i.e., coated with antibodies, PEG or sylane). Furthermore, by spanning among all the flow cells employed, it was possible to finely choose the design, which better achieved the requirements of the experiment. For example, MT experiments requiring a fast buffer exchange and low perturbation could take advantage of wider flow cell, while experiments with a scarce specimen would prefer the use of a flow cell with a volume as small as possible. The flow cell dimensions presented here were adequate for fluorescence measurements, while the high drag forces achieved perturbed MT experiments. Altogether, these results provide a strong basis for the custom-design of home-fabricated microfluidics, providing a guide for the design and fabrication of flow cells suitable for single-molecule studies.

## Methods

The datasets generated during and/or analyzed during the current study are available from the corresponding author on reasonable request.

### Combined lateral MT-TIRF apparatus

Experiments were performed in a MT-TIRF setup similar to a one described before^33^. In brief, a 488 nm laser source (Vortran Stradus) was focused on the back focal plane of a high numerical aperture objective (Olympus UAPON TIRF 100 ×). Two separate detectors were used to visualize the emission of the fluorophores in the sample and the magnetic beads; an EM-CCD temperature-controlled camera (Andor Ixon Ultra 888) and a CCD or CMOS camera (Pulnix 6710CL/Mikrotron MC1362) for bright-field video microscopy. The MT setup was controlled by a custom-written code in LabVIEW 2011. The fluorescence camera was controlled by the Andor Solis software (Solis version 4.30.30036.0; SDK version 2.102.33036.0; www.andor.com).

### Flow cells

Glass coverslips (Menzel-Gläser, #1) were cleaned by 30 min sonication in acetone followed by 30 min in isopropanol, and dried using compressed air. The clean surface was coated with 1% polystyrene dissolved in toluene. The top cover glass contained three holes drilled with a laser engraver, as well as two-inlet paraffin wax (Parafilm M, Bernis USA) gaskets (VLS2.30, Universal Laser Systems). The two cover glass slides and the gasket were sandwiched and heated up for a few seconds at 120 °C to assemble the flow cell. The cells were then incubated with an Antidigoxigenin (25 ng/μl) solution (Roche) overnight at 4 °C and were passivated for at least 2 h using BSA (NEB). The cells were stored in a humid and sealed container at 4 °C until further use.

### DNA substrates (λ)

λ molecules were fabricated as described elsewhere^51^. Briefly, the CosR-tail oligonucleotide was biotin tailed and the CosL-tail oligonucleotide was digoxigenin tailed using Terminal Transferase (NEB) and either BIO-dUTP or DIG-dUTP (Roche). The modified oligonucleotides were purified using a Qiaquick nucleotide removal kit (Qiagen). The tailed oligonucleotides were subsequently annealed and ligated to λ DNA (NEB) overnight using T4 DNA ligase (NEB).

λ/2 molecules were fabricated as described elsewhere^33,51^. N6-Mehtyladenine-free λ DNA (NEB) was cleaved with XbaI, giving two ~ 24 kbp fragments. These fragments and the aforementioned tailed oligonucleotides in addition to the XbaI-B oligonucleotide were subsequently annealed and ligated overnight using T4 DNA ligase (NEB).

### Fluorescein multichannel laminar-flow experiments for rapid buffer exchange

To facilitate focusing on the surface, a very diluted sample of 1 µm-sized paramagnetic beads (Dynabeads) was flowed inside the flow cell prior to the measurements in MilliQ water. The specimen was abundantly cleaned with water before performing the fluorescence measurements. Fluorescein (Sigma Aldrich) was dissolved in 50 mM Hepes pH 8 at a final concentration of 1 mM. To achieve boundary shifting measurements, the fluorescein reservoir was intermittently switched with the buffer reservoir by using two automated syringe pumps (neMESYS). The syringes were controlled with the neMESYS UserInterface software (version 2014.3.10.3; www.cetoni.de). Briefly, a square-wave pattern was set for syringes to alternate flow-rates, keeping a constant flow-rate in the central channel (Q from 7 to 200 µl min^−1^). Fluorescence images were acquired using the Andor Solis software, at a frequency of 9.52 Hz, using a EM level of 100 and cooling the sensor to − 60 °C. 500 frames were acquired for each tested flow rate. All measurements were carried out in the central channel of the flow cells, approximately at 1–2 mm from the two inlets intersection. Data analysis was performed using the Andor Solis software, followed by ImageJ and Origin. For each flow rate, at least 5 regions of interest (ROI) of 50 × 50 pixels were selected in a homogeneous area and their integrated fluorescence intensity was analyzed in Origin.

### DNA-tethering multichannel laminar-flow experiments

Tethers of λ DNA were obtained by flowing DNA molecules in a buffer containing 50 mM Tris pH 7.5, 100 mM KCl, 2.5 mM MgCl_2_, 1 mM DTT and 0.1 mg ml^−1^ BSA. After binding, unbound DNA molecules were extensively washed away. To visualize DNA molecules under the TIRF illumination, Sytox Green was used. DNA molecules were stained with 100 nM Sytox Green in standard buffer supplemented with 1 mM Trolox, 20 mM glucose, 8 µg/ml glucose oxidase and 20 µg/ml catalase to minimize photobleaching. Fluorescence images were acquired using the same settings described in the previous section. At least 4000 frames were recorded. neMESYS syringes were operated in the same way, keeping the flow-rate in the central channel constant (Q to 10–200 µl min^−1^). Data analysis was performed using Andor Solis and Origin as described in^42^. For each DNA molecule of interest, a main region of interest (ROI) was selected around the DNA molecule (additional ROIs around the main ROI were also selected for background correction). Integrated fluorescence intensities were then analyzed in Origin.

### Flow stretch force calibration

Tethers of λ/2 DNA molecules were obtained by mixing DNA with 1 µm-sized magnetic beads (Dynabeads, MyOne streptavidin, Invitrogen) in a buffer containing 10 mM PB (pH 7), 10 mM NaN_3_, 0.2 mg ml^−1^ BSA, and 0.1% Tween 20. The sample was then injected in an Antidigoxigenin functionalized 1 parafilm layer and 1 mm wide flow cell and incubated for 10–15 min before applying the external flow. In a preliminary step, each DNA molecule was individually calibrated using a standard MT force calibration protocol^50^. Then, the magnets were removed and the flow was initialized by using the two in-parallel-working automatized syringe pumps filled with the same buffer. Briefly, a step-like profile with increasing flow rates (from 2 to 150 µl min^−1^, 5 s for each step) was initialized in the neMESYS software. The 3D position of each bead was recorded with our custom-written LabVIEW code.

### Correlated MT and TIRF measurements using Sytox Green

Tethers of λ/2 DNA molecules were obtained following the same procedure than for the flow stretch MT experiments, in the same buffer. The force applied with the magnets to the tethered DNA was 1 pN. Then, a flow was applied by using the two in-parallel-working automatized syringe pumps. One syringe pump was filled with buffer and the other contained the same buffer supplemented with 50 nM Sytox Green, 1 mM Trolox, 20 mM glucose, 8 µg/ml glucose oxidase and 20 µg/ml catalase. 500 frames were acquired while the synchronized syringe pumps alternated between 20 and 180 µl min^−1^, so the DNA located in the middle part of the channel was exposed to buffer and buffer containing Sytox Green at a constant 200 µl min^−1^ flow rate. At the same time, the tracking of the beads was followed by videomicroscopy at 120 Hz. Analysis of the bead’s spatial coordinates and fluorescence data was performed as described in previous sections.

## Supplementary information

Supplementary file1
