# Supplementary material for: Characterizing microfluidic approaches for a fast and efficient reagent exchange in single-molecule studies
Source: Sci Rep. 2020 Oct 22;10:18069. doi: 10.1038/s41598-020-74523-w (PMC7581773; doi:10.1038/s41598-020-74523-w)
Supplement: Supplementary file 1 — Supplementary file1 [file 41598_2020_74523_MOESM1_ESM.docx]

Supplementary Information

**Characterizing microfluidic approaches for a fast and efficient reagent exchange in single-molecule studies**

*Julene Madariaga-Marcos^1^, Roberta Corti^2,3^, Silvia Hormeño^1^ and Fernando Moreno-Herrero^1*^.*

^1^ Department of Macromolecular Structures, Centro Nacional de Biotecnología, Consejo Superior de Investigaciones Científicas, Madrid, Spain

^2^ School of Medicine & Surgery, University of Milano-Bicocca, Monza, Italy

^3^ Department of Materials Science, University of Milano-Bicocca, Milan, Italy

* Corresponding author: [fernando.moreno@cnb.csic.es](mailto:fernando.moreno@cnb.csic.es)

**Supplementary Methods:**

***Force calibration in Flow Stretch experiments:***

We have previously characterized the force exerted by the flow on a tethered bead in the absence of a magnetic force [1]. In such a flow stretch experiment, each DNA molecule is elongated along the surface by the force of the flow. We took advantage of MT by precisely measuring the extension of the tether by tracking the bead with the standard CCD camera and software. We employed both syringes of the neMESYS system in parallel to achieve a final flow rate of 5 - 50 µl min^-1^. Extension versus flow data (**Supplementary Figure 3A**) could be correlated with the applied force using previously taken force-extension curves performed with the vertical MT setup as described in [1].

Briefly, under flow stretch, the bead suffers a drag force given by Stokes' Law:

$F_{drag}= 6\pi R\eta v_{flow}$ (S1)

where $v_{flow}$ is the linear velocity of the flow at the vicinity of the bead, $R$ the radius of the bead and $\eta$ the viscosity of the fluid. The linear trend given by Stokes' Law was experimentally observed (**Suplementary Figure 3B**).

The linear velocity of the flow can be expressed as a fraction of the maximum velocity at the center of the channel (**Equation S2**), which is defined as $v_{max}=2\cdot v_{mean}=\frac{2\cdot Q}{w\cdot d}$ [2] being $d$ and $w$ the channel height and width, respectively. In our case, $d\approx$100 μm and $w\approx$ 1 mm, giving a cross section of the cell of 0.1 mm^2^.

$v_{flow}=k v_{max}=$ $\frac{2\cdot Q}{w\cdot d} k$ (S2)

The viscosity, $\eta$, should be corrected because the radius of the bead is comparable to the distance of the bead to the surface following [3]. Therefore, $\eta^{*}=1.6 \eta$.

At equilibrium, $F_{WLC}={F_{drag}}/{\cos\alpha\approx F_{drag}}$, for $\alpha\to0$. We can then estimate the linear velocity of the flow in the proximity of the bead, by fitting **Equation S3** and **Equation S4** to the extension data (**Suplementary Figure 3A**) and the force data (**Suplementary Figure 3B**).

$l\left( Q \right)= L \left( 1-{\frac{1}{2}\left( \frac{k_{B}T}{P\cdot6\pi R\eta^{*}\frac{2\cdot Q}{w\cdot d} k} \right)}^{1/2} \right)$ (S3)

$F\left( Q \right)= 6\pi R\eta^{*}\frac{2\cdot Q}{w\cdot d} k$ (S4)

Assuming a persistence length of 40 nm, we obtained a contour length of $L$ = 8.36 µm, close to the expected crystallographic length of the molecule. The obtained forces (**Suplementary Figure 3B**) were higher than those measured in conventional flow cells (4 pN compared to 1.5 pN), as expected from the higher linear velocities, and data nicely fitted the Stokes drag equation. The obtained linear velocities are $v_{flow}=0.017 v_{max}$ (1.7% of $v_{max}$) from the fitting to the extension data, and $v_{flow}=0.018 v_{max}$(1.8% of $v_{max}$) from the fitting to the force data.

**Supplementary References**

[1] Madariaga-Marcos, J. *et al.* Force determination in lateral magnetic tweezers combined with TIRF microscopy. *Nanoscale* **10**, 4579–4590 (2018).

[2] Çengel, Y. A. & Cimbala, J. M. *Fluid mechanics fundamentals and applications* (McGraw Hill, 2006).

[3] Kruithof, M., Chien, F., de Jager, M. & van Noort, J. Subpiconewton dynamic force spectroscopy using magnetic tweezers. *Biophysical Journal* **94**, 2343–2348 (2008).

| **Final Q**  **(µl min^-1^)** | **Maximum Q - Q_max_**  **(µl min^-1^)** | **Minimum Q – Q_min_**  **(µl min^-1^)** |
| --- | --- | --- |
| 200 | 190 | 10 |
| 100 | 90 | 10 |
| 50_1_ | 40 | 10 |
| 50_2_ | 45 | 5 |
| 40 | 35 | 5 |
| 30 | 25 | 5 |
| 20_1_ | 15 | 5 |
| 20_2_ | 17 | 3 |
| 15 | 12 | 3 |
| 10 | 7 | 3 |
| 7 | 5 | 2 |

**Supplementary Table 1. Final flow rates Q obtained as the sum of Q_max_ and Q_min_.** For flow rates Q = 50 µl min^-1^ and Q = 20 µl min^-1^ two different flow rate ratios have been employed, showing very similar results. For flow cells with high cross section (3A and 4A), a complete boundary exchange was not achieved for the lowest flow rates.

| **Flow cell** | **Cross section**  **(µm^2^)** | **y0**  **(m)** | **D**  **(m^2^/s)** | **a**  **(m)** |
| --- | --- | --- | --- | --- |
| 1 mm - 1 layer | A | (8.7 ± 0.8) · 10^-4^ | (2 ± 1) · 10^-9^ | (6 ± 1) · 10^-5^ |
| 2 mm - 1 layer | 2A | (2 ± 1) · 10^-4^ | (1 ± 2) · 10^-10^ | (1.3 ± 0.9)· 10^-4^ |
| 1 mm - 2 layer | 2A | (6 ± 2) ·10^-4^ | (1.5 ± 0.6) · 10^-9^ | (1 ± 1) · 10^-4^ |
| 1.5 mm - 2 layer | 3A | (1.9 ± 0.2) · 10^-4^ | (5.1 ± 0.8) · 10^-10^ | (1.0 ± 0.9) · 10^-5^ |
| 2 mm - 2 layer | 4A | (1 ± 2) · 10^-4^ | (3 ± 2) · 10^-9^ | (4 ± 2) · 10^-4^ |
| *Theoretical* |  | 10^-3^ | 4.9 · 10^-10^ | 1-2 · 10^-4^ |

**Supplementary Table 2. Independent Taylor-Aris fit for the five considered flow cells.** Four free parameters were employed: y_0_ (distance from the inlet), v (the fluid speed, not reported), a (channel height) and D (diffusion constant). Data displayed here are the average of independent fits.

| **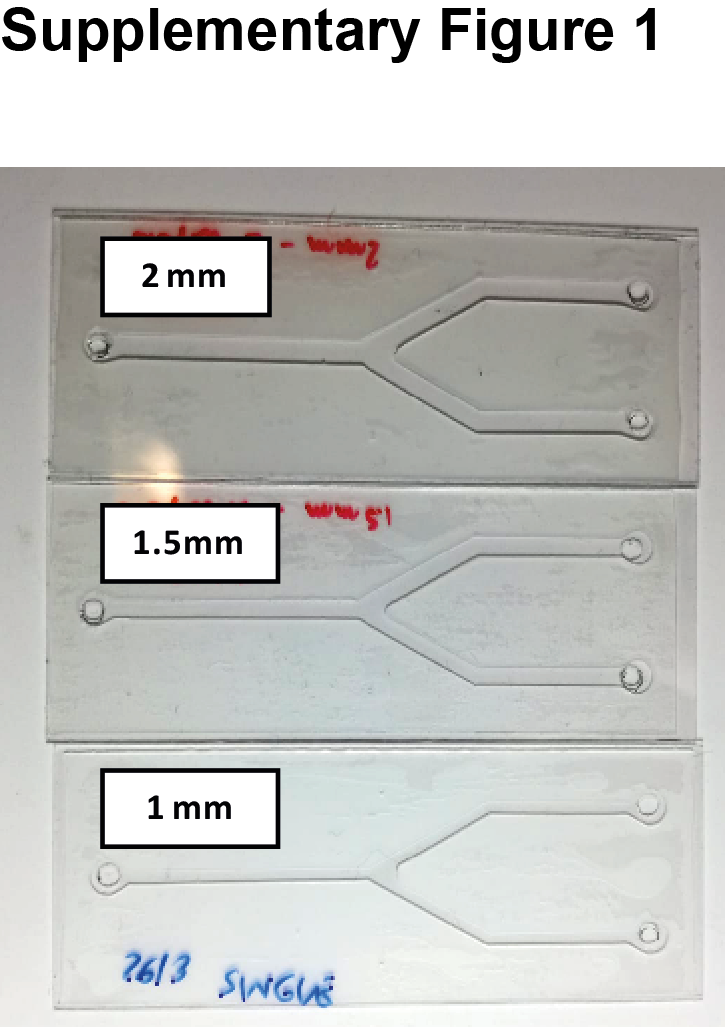** |
| --- |
| **Supplementary Figure 1. Picture of the different flow rate channel widths employed in this study.** |

| 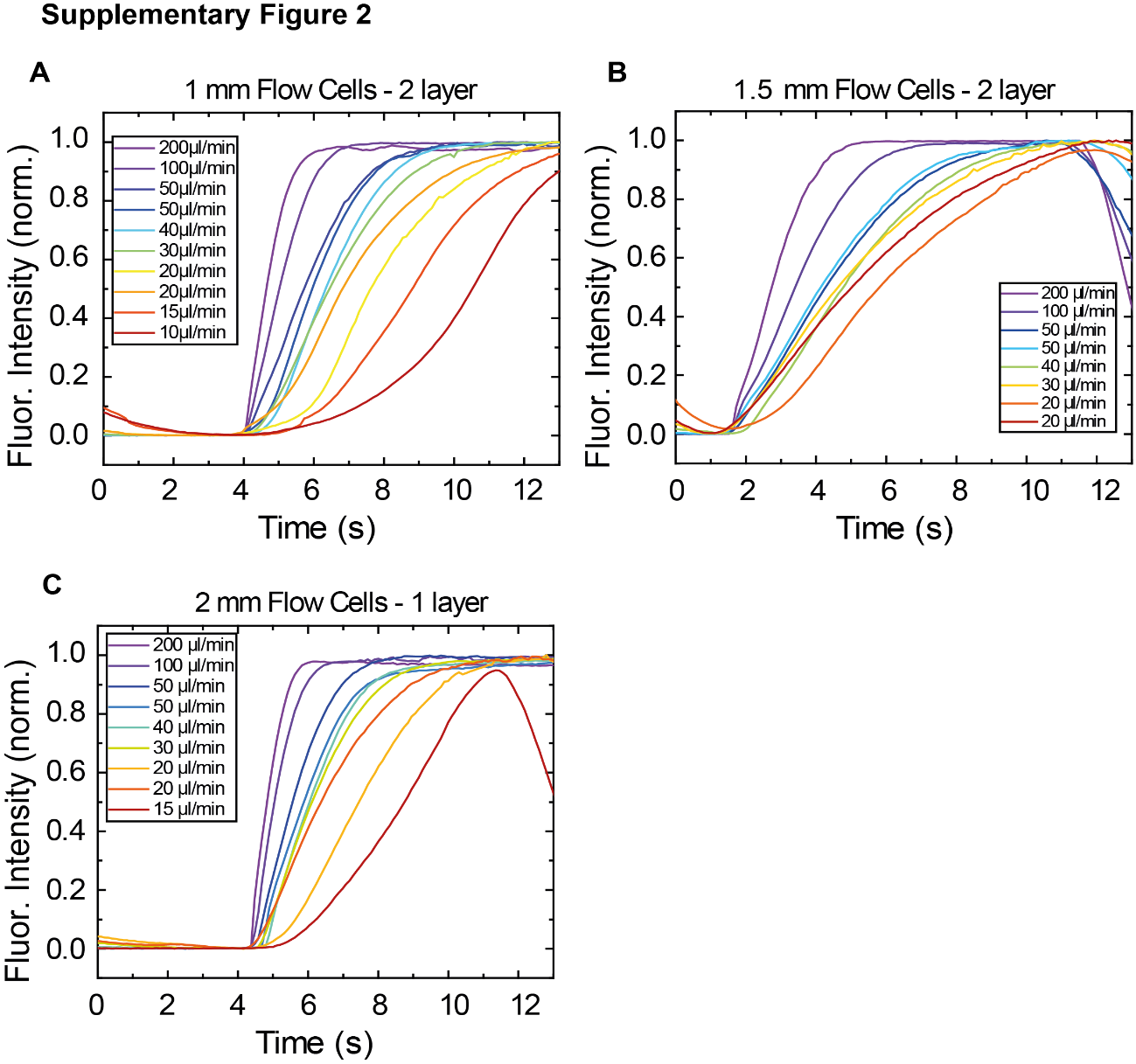 |
| --- |
| **Supplementary Figure 2. Measured changes in fluorescence intensity at different flow rates for all the flow cells employed in this study.**  (A) Data for 1 mm wide double layer flow cells.  (B) Data for 1.5 mm wide double layer flow cells. (C) Data for 2 mm wide single layer flow cells. |

| **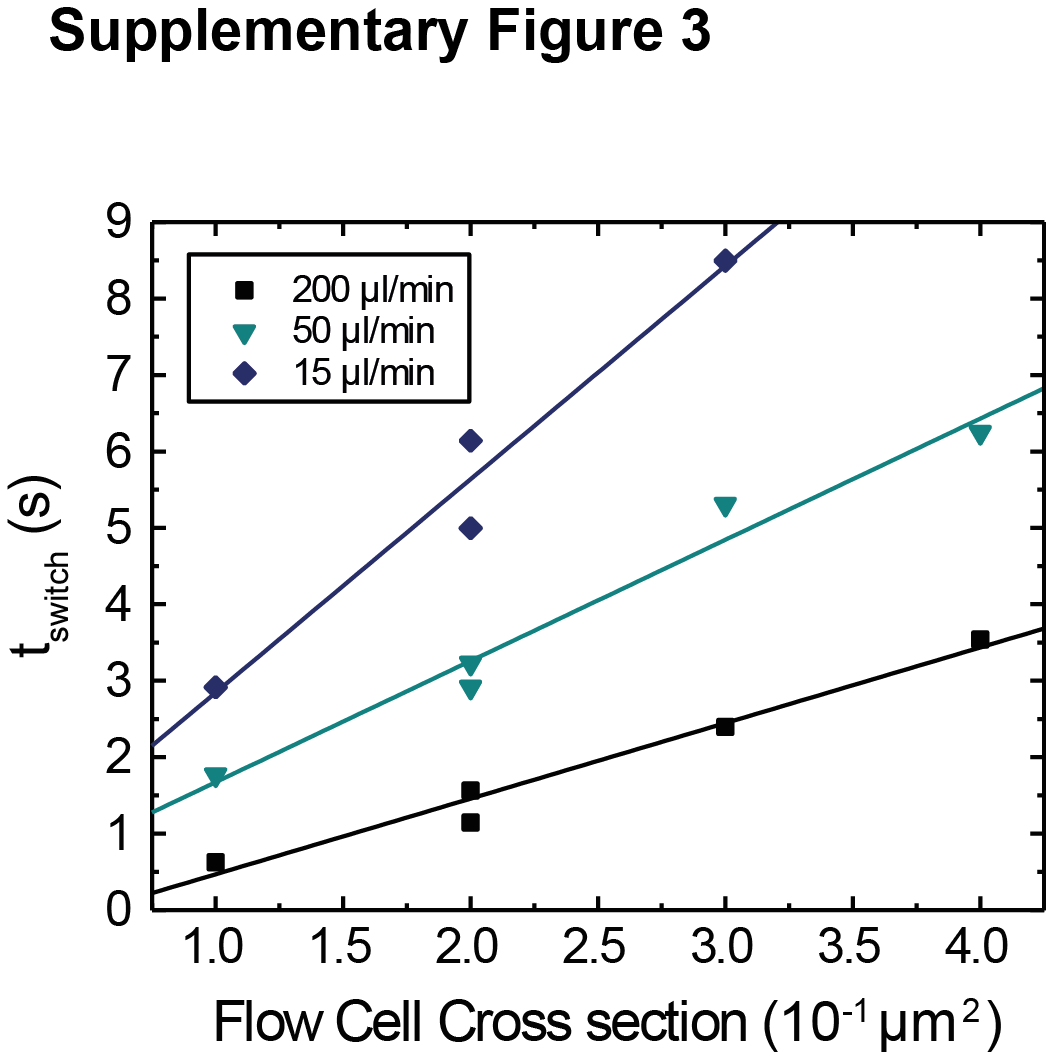** |
| --- |
| **Supplementary Figure 3. The boundary shifting time as a function of the flow cell cross section for different flow rates.** |

| 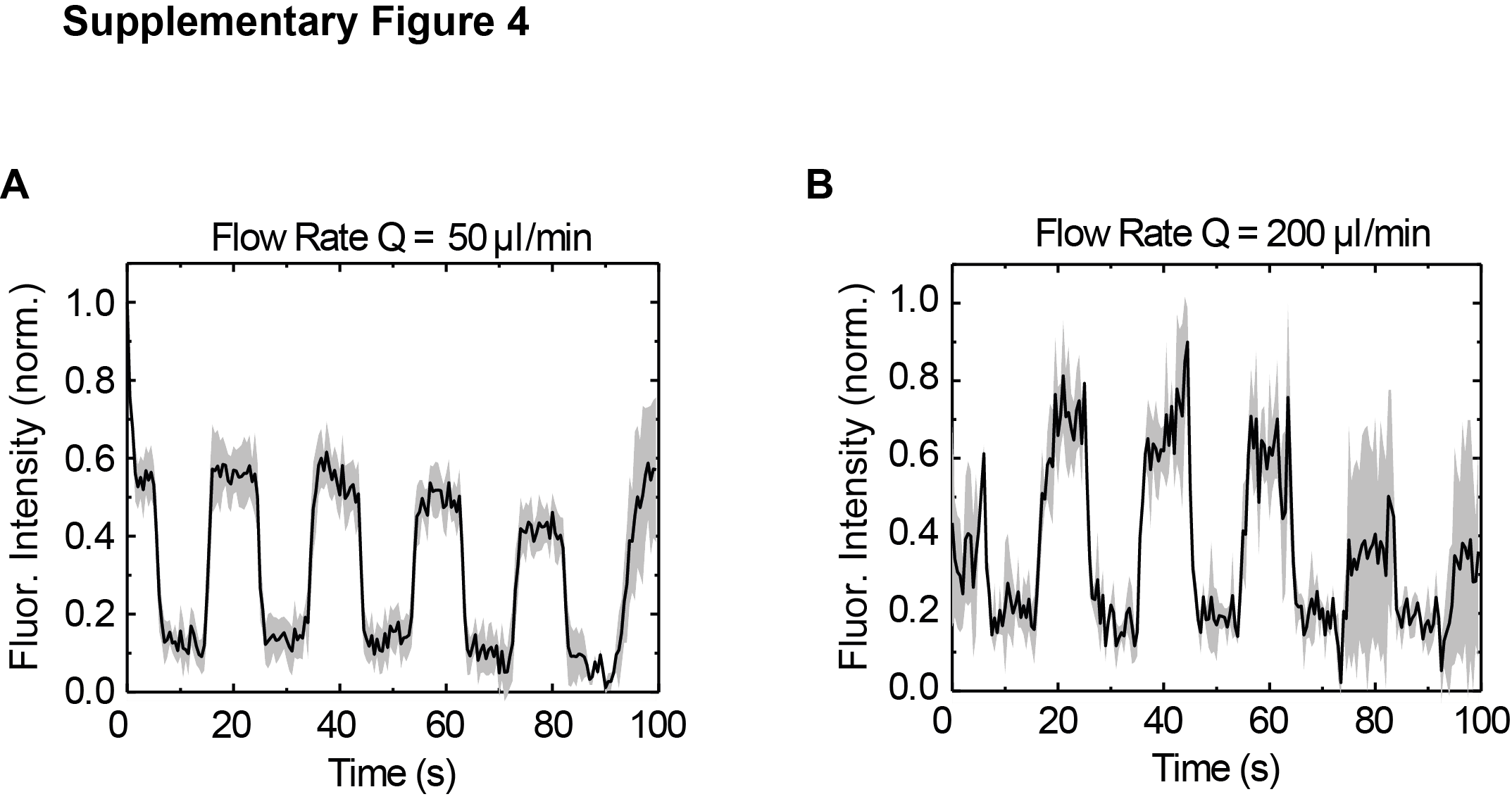 |
| --- |
| **Supplementary Figure 4. Measured changes in fluorescence intensity at different flow rates upon the introduction of Sytox Green.**  (A) Data for measurements performed at 50 µl min^-1^. Data have an average standard deviation of 0.07.  (B) Data for measurements performed at 200 µl min^-1^. Data have an average standard deviation of 0.1. The decrease in intensity for the last cycles is due to the breakage of several DNA molecules under laser exposure. |

| **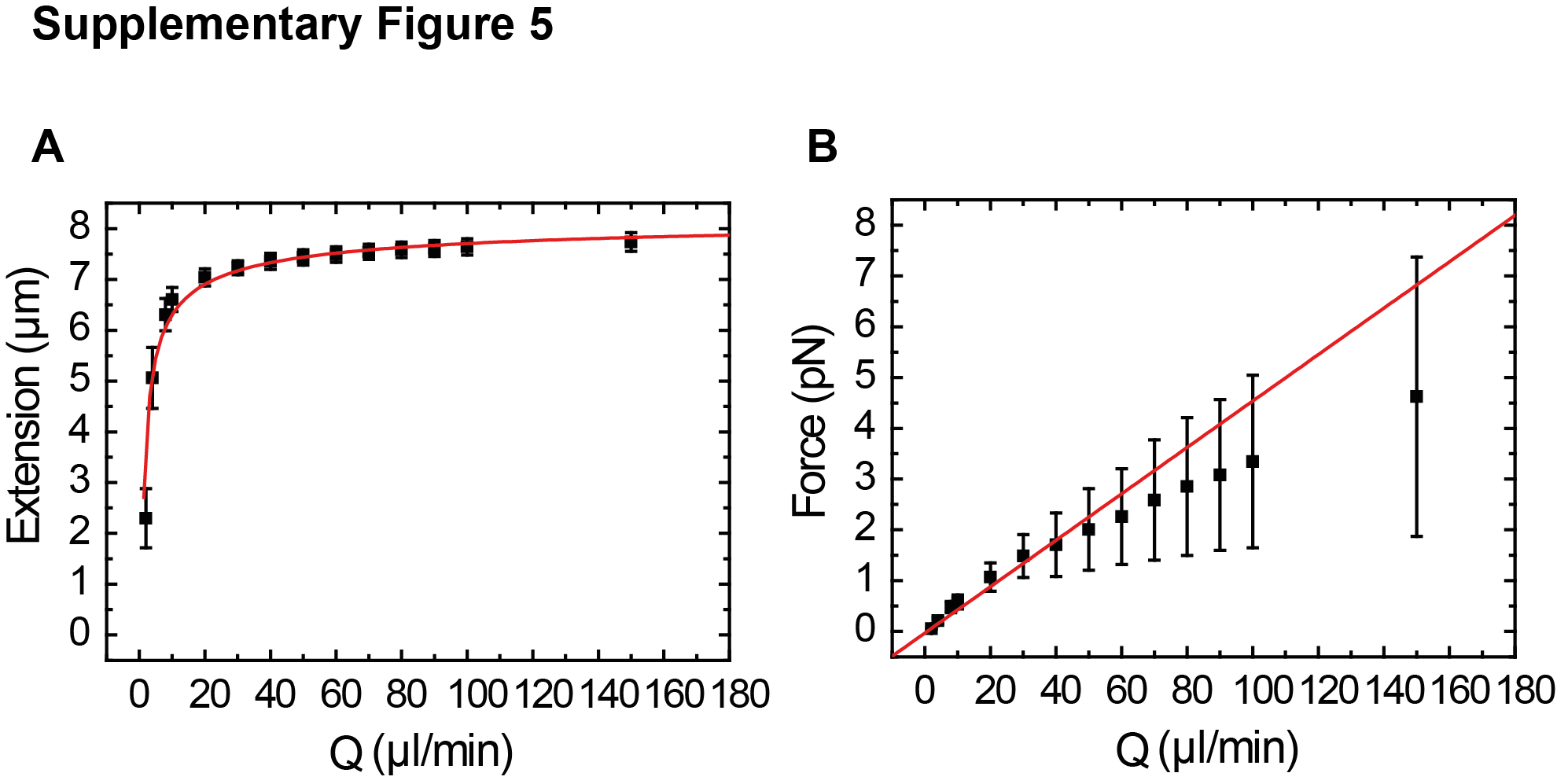** |
| --- |
| **Supplementary Figure 5. Extension and force as a function of the flow rate.**  (A) DNA extension as a function of the flow rate. The data was fitted using force-extension curves taken with MT and an inverse Worm-Like Chain model.  (B) Mean force on the bead as a function of flow rate was described by Stokes’ drag. It increased linearly up to a maximum value of ~4 pN. |
